# Benefits of Fasting Abbreviation with Carbohydrates and Omega-3 Infusion During CABG: a Double-Blind Controlled Randomized Trial

**DOI:** 10.21470/1678-9741-2018-0336

**Published:** 2019

**Authors:** Gibran Roder Feguri, Paulo Ruiz Lúcio de Lima, Anna Carolina Franco, Felipe Ramos Honorato De La Cruz, Danilo Cerqueira Borges, Laura Ramos Toledo, Neuber José Segri, José Eduardo de Aguilar-Nascimento

**Affiliations:** 1 Department of Cardiology and Cardiovascular Surgery, Hospital Geral Universitário, Universidade de Cuiabá (HGU-UNIC), Cuiabá, MT, Brazil.; 2 Department of Cardiovascular Surgery, Hospital Geral Universitário, Universidade de Cuiabá (HGU-UNIC), Cuiabá, MT, Brazil.; 3 Department of Physical Therapy, Hospital Geral Universitário, Universidade de Cuiabá (HGU-UNIC), Cuiabá, MT, Brazil.; 4 Department of Statistics, Universidade Federal do Mato Grosso (UFMT), Cuiabá, MT, Brazil.; 5 Universidade Federal do Mato Grosso (UFMT), Cuiabá, MT, Brazil.; 6 Universidade de Várzea Grande (UNIVAG), Várzea Grande, MT, Brazil.

**Keywords:** Myocardial Revascularization, Perioperative Care, Atrial Fibrillation, Inflammation, Hyperglycemia

## Abstract

**Objective:**

To assess postoperative clinical data considering the association of preoperative fasting with carbohydrate (CHO) loading and intraoperative infusion of omega-3 polyunsaturated fatty acids (ω-3 PUFA).

**Methods:**

57 patients undergoing coronary artery bypass grafting (CABG) were randomly assigned to receive 12.5% maltodextrin (200 mL, 2 h before anesthesia), (CHO, n=14); water (200 mL, 2 h before anesthesia), (control, n=14); 12.5% maltodextrin (200 mL, 2 h before anesthesia) plus intraoperative infusion of ω-3 PUFA (0.2 g/kg), (CHO+W3, n=15); or water (200 mL, 2 h before anesthesia) plus intraoperative infusion of ω-3 PUFA (0.2 g/kg), (W3, n=14). The need for vasoactive drugs was analyzed, in addition to postoperative inflammation and metabolic control.

**Results:**

There were two deaths (3.5%). Patients in CHO groups presented a lower incidence of hospital infection (RR=0.29, 95% CI 0.09-0.94; *P*=0.023), needed fewer vasoactive drugs during surgery and ICU stay (*P*<0.05); and had better blood glucose levels in the first six hours of recovery (*P*=0.015), requiring less exogenous insulin (*P*=0.018). Incidence of postoperative atrial fibrillation (POAF) varied significantly among groups (*P*=0.009). Subjects who receive ω-3 PUFA groups had fewer occurrences of POAF (RR=4.83, 95% CI 1.56-15.02; *P*=0.001). Patients in the W3 group had lower ultrasensitive-CRP levels at 36 h postoperatively (*P*=0.008). Interleukin-10 levels varied among groups (*P*=0.013), with the highest levels observed in the postoperative of patients who received intraoperative infusion of ω-3 PUFA (*P*=0.049).

**Conclusion:**

Fasting abbreviation with carbohydrate loading and intraoperative infusion of ω-3 PUFA is safe and supports faster postoperative recovery in patients undergoing on-pump CABG.

**Table:** 

Abbreviations, acronyms & symbols				
ACERTO	= ACEleração da Recuperação TOtal Pós-operatória		CRP	= C-reactive protein
AMI	= Acute myocardial infarction		CVA	= Cerebrovascular accident
ANOVA	= Analysis of variance		ICU	= Intensive care unit
BMI	= Body mass index		LVEF	= Left ventricular ejection fraction
CABG	= Coronary artery bypass grafting		POAF	= Postoperative atrial fibrillation
CAD	= Coronary artery disease		SGA	= Subjective global assessment
CC	= Cross-clamp		SIRS	= Systemic inflammatory response syndrome
CHO	= Carbohydrate		W3	= Group W3
CPB	= Cardiopulmonary bypass		ω-3 PUFA	= Omega-3 polyunsaturated fatty acids

## INTRODUCTION

Advances in medicine and questioning of obsolete practices have led to a search for factors that could improve the quality of healthcare and minimize errors. For this purpose, surgical services have created multiprofessional and multidisciplinary teams and established fast-track and checklist protocols to determine perioperative care associated with lower morbidity and mortality, reduced costs, and faster postoperative recovery^[1]^.

The ACERTO Project (*ACEleração da Recuperação TOtal Pós-operatória*) is an example of a national multimodal protocol aimed at accelerating postoperative recovery of patients. The basis for this protocol include the abbreviation of preoperative fasting with intake of carbohydrate-rich fluids; early postoperative feeding and mobility; reduced length of stay in the intensive care unit (ICU); perioperative nutritional assessment and therapy; rational antibiotic prophylaxis; restriction of perioperative intravenous fluids; reduced use of drains and catheters; sedation; and informed consent^[2,3]^.

It is estimated that 17.5 million people die every year from cardiovascular diseases, which represents 31% of all deaths globally. Out of those, approximately 7.4 million (42%) are the result of coronary artery disease (CAD)^[4]^. A therapeutic option for CAD and one of the most studied and performed surgeries in the world^[5]^, coronary artery bypass grafting (CABG) reestablishes blood flow in obstructed coronary vessels. This procedure is largely carried out with the support of cardiopulmonary bypass (CPB). Nevertheless, the use of CPB has been associated with both the onset and worsening of systemic inflammatory response syndrome (SIRS), which occurs in the postoperative period of cardiovascular surgery. SIRS is characterized by leukocytosis, release of proinflammatory cytokines (among other proteins in the acute phase), and increased vascular permeability, resulting in accumulation of interstitial fluid and organic lesions mainly in the heart, kidneys, and lungs, thereby contributing to increased operative morbidity^[6,7]^.

Despite being controversial, corticosteroid use can interfere with adverse effects resulting from patient's blood going through the CPB circuit. Still, the literature corroborates the hypothesis that it is necessary to act on several related factors to relieve the inflammation caused by CPB^[7,8]^. It is in this context that immunomodulatory drugs, such as omega-3 (ω-3), have gained prominence in cardiovascular surgery with promising outcomes^[9]^.

Omega-3 polyunsaturated fatty acids (ω-3 PUFA) are directly involved in the balance and metabolism of body fat, immunity, and postoperative immune response^[10]^. Evidence indicates that ω-3 PUFA can be beneficial for patients undergoing cardiovascular surgery as it acts in the postoperative inflammatory response^[11]^. A recent meta-analysis showed that ω-3 can reduce the incidence of postoperative atrial fibrillation (POAF) as well as length of hospital stay^[12]^. Although this type of oil can be administered orally-because it is rapidly absorbed by cell membranes-it can also be given intravenously, particularly for patients in the ICU and a number of surgical procedures^[10]^.

POAF is among the most common complications after CABG, with an incidence ranging from 10% to 40%. This type of postoperative arrhythmia can interfere with the clinical evolution of patients following CABG, leading to increased costs, higher morbidity, longer hospital stays, and even mortality. Perioperative strategies to mitigate POAF are highly important to cardiovascular surgery and thus are being constantly studied^[13,14]^.

On the other hand, abbreviation of preoperative fasting with oral intake of carbohydrates (CHO) can minimize insulin resistance and hyperglycemia after cardiovascular surgery^[15,16]^. Moreover, it can also lower the incidence of postoperative nausea and vomiting^[17]^. Due to its benefits, this has become a practice accepted by several anesthesiology societies^[18]^. In particular for cardiovascular surgery, randomized studies have shown that brief fasting with CHO intake two hours before induction of anesthesia can minimize the need for perioperative vasoactive drugs and reduce length of ICU and hospital stay^[15,16]^.

Within this framework, this study set out to assess whether abbreviation of fasting with CHO-rich fluids associated with intraoperative infusion of ω-3 PUFA could interfere with postoperative morbidity, control of blood glucose and inflammation, as well as recovery in patients undergoing on-pump CABG.

## METHODS

This is a prospective, randomized, double-blind clinical trial, which evaluated patients undergoing on-pump CABG at General University Hospital (Cuiabá/MT - Brazil) between March 2014 and June 2016. The study was approved by the Ethics Research Committee of Universidade de Cuiabá (UNIC) under CAAE No. 30493514.5.000.5165, on June 3, 2014. The study was registered on ClinicalTrials.gov under NCT: 03017001.

Male and female patients aged between 18 and 80 years with CAD and eligible for elective on-pump CABG were included. All patients signed an informed consent form. Exclusion criteria included: patients under 18 years or older than 80 years; those who did not give their informed consent; insulin-dependent diabetes; fasting blood glucose level above 150 mg/dL; patients with gastroparesis or gastroesophageal reflux disease; heavy use of ω-3 PUFA or corticosteroids up to six months prior to surgery; patients with cirrhosis of the liver (Child class A, B or C); acute or chronic kidney injury with creatinine equal to or greater than 2.0; patients on hemodialysis; uncontrolled dyslipidemia (triglycerides twice as high as the reference value); previously diagnosed coagulopathy and/or platelet count lower than the reference value; allergy to fish or shrimp; emergency surgery; reoperation; combined surgical procedures; patients with acute coronary syndrome and mechanical complications of myocardial infarction; subjective global assessment (SGA) class C; any type of transfusion three months prior to surgery.

Sixty patients with CAD were included consecutively and randomly assigned (1:1) to four groups with 15 patients each, using QuickCalcs(tm) (GraphPad Software Inc., San Diego, CA, USA) online software. Interventions were defined as follows: 1) Control group, brief fasting with 200 mL of water two hours before surgery; 2) CHO group, brief fasting with oral intake of 200 mL of water with 25 g (12.5%) of maltodextrin two hours before surgery; 3) CHO+W3 group, brief fasting with 200 mL of water with 25 g (12.5%) of maltodextrin two hours before surgery and intraoperative infusion of 0.2 g/kg of ω-3 PUFA for four hours; and 4) W3 group, brief fasting with 200 mL of water and intraoperative infusion of 0.2 g/kg ω-3 PUFA for four hours.

A hospital dietitian, as the only attendant to do the charted randomization, directed the preoperative intake. The drinks delivered by the ward nurse were given to the patients prior to transportation to the operating room. The anesthesiologist was also informed (by the dietician) of which patients would receive intraoperative ω-3 PUFA. None of the surgical team (surgeon or assistants) was aware of the patient's assignments. A team of cardiologists and intensive care physicians, also blind to study design and patient randomization, collected all data.

### Variables Analyzed

The main outcome variables analyzed in the study included: incidence of POAF; infection; major combined cardiovascular events - acute myocardial infarction (AMI), cerebrovascular accident (CVA), and hospital mortality; need for vasoactive drugs in the operating room and in the ICU; blood sugar level, monitored through capillary blood glucose serial testing; assessment of insulin resistance based on the amount of exogenous insulin used to maintain glycemia at <150 mg/dL intraoperatively and in the first six hours of recovery in the ICU; evaluation of the inflammatory response through acute phase inflammatory markers; ultrasensitive C-reactive protein (CRP); and interleukins 6 and 10 (IL-6 and IL-10).

The secondary outcome variables analyzed in the study included: bronchial aspiration during induction of anesthesia; bleeding in the first 12 hours of recovery in the ICU; perioperative use of packed red blood cells and/or blood components; length of stay in the ICU; duration of mechanical ventilation; and length of postoperative hospital stay.

### Anesthesia and Surgical Technique

The same surgeon carried out all the surgical procedures. Routine anesthetic practices were used. Induction was performed with 0.2 mL/kg etomidate infusion, 5 mcg/kg fentanyl citrate, and 0.1 mg/kg pancuronium bromide. Anesthesia was maintained through isoflurane inhalation at a normal dose (0.6-1.8%) and, if needed, bolus administration of 2 mcg/kg fentanyl, 0.1 mg/kg midazolam, and 0.05 mg/kg pancuronium bromide to induce muscle relaxation. Patients were kept on mechanical ventilation with oxygen (FiO_2_) at 60% (or higher, as needed).

Standard surgical technique was used, and the inlet through median sternotomy with CPB in the allotted time. A membrane oxygenator (Braile Biomédica, São José do Rio Preto, Brazil) was used. For myocardial protection, hypothermic intermittent anterograde blood cardioplegia (every 15-20 min) and mild systemic hypothermia (33-35°C) were used. Patients received 1.5 g of intravenous cefuroxime one hour before anesthetic induction and one additional dose after CPB. Maintenance doses (750 mg) were administered every six hours for 48 hours. Routine 7 mg/kg of methylprednisolone was administered intravenously during anesthetic induction.

### Blood Sample Collection for Analysis

**Fig. 1:**
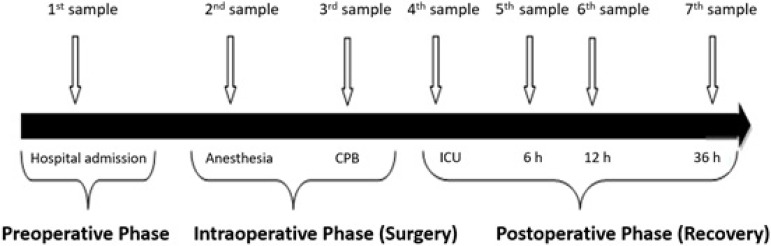
Graphical representation of seven blood samples collected for analysis in different phases of the perioperative period.

Except for the first blood sample, collection was performed through the invasive blood pressure circuit. Seven blood samples were collected at different times, as illustrated in Figure 1.

### Statistical Analysis

Sample calculation was performed, and every variable was submitted to the Shapiro-Wilk test for normality before analysis. For values with Gaussian distribution, paired or unpaired t-tests and analysis of variance (ANOVA) for repeated measures were performed; non-parametric tests of Friedman, Wilcoxon, Mann-Whitney, and Kruskal-Wallis were used for non-Gaussian data. Qualitative variables were analyzed using chi-square and Fisher's exact tests. Descriptive analyses were performed on Microsoft Office Excel 2007 (Microsoft Corp., Redmond, WA, USA) and statistical analyses were performed with Stata v.13. (Stata Statistical Software: Release 13. College Station, TX: StataCorp LP, USA). Every test was two-tailed at 80% power, with significance level set at *P*<0.05 (α=5%).

## RESULTS

**Fig. 2:**
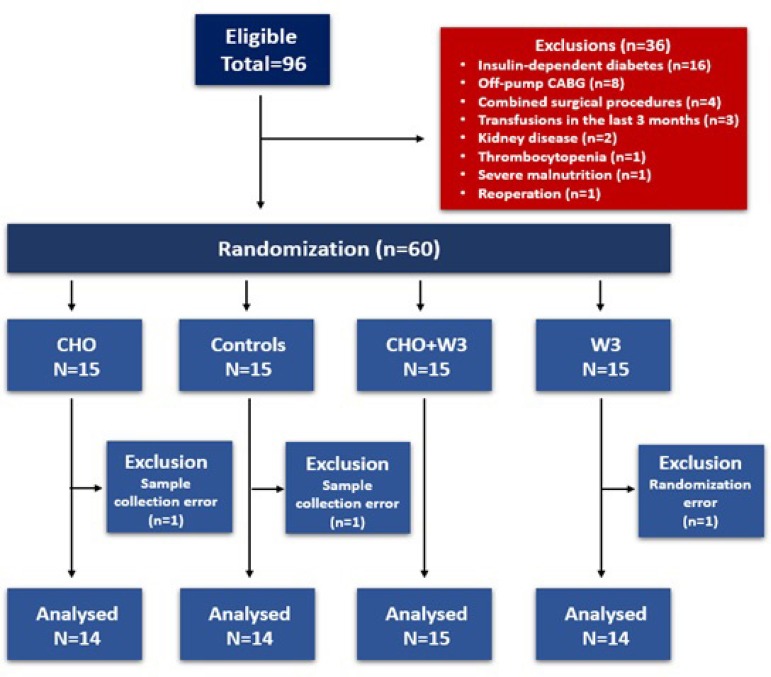
Flowchart showing patient selection for the study.

The study design is shown in the flowchart in Figure 2. Of the 96 eligible patients, 36 were excluded for different reasons. The remaining 60 patients were randomly assigned into four groups. After exclusion of one patient in each of the CHO, W3, and Control groups, 57 patients were analyzed (CHO+W3: n=15; others: n=14).

Anthropometric data per group are shown in Table 1 and clinical data, in Table 2. Analyses of the variables listed in the aforementioned tables showed that the groups are homogenous.

**Table 1:** Demographic and anthropometric data of 57 patients undergoing on-pump CABG.

	CHO (n=14)	Control (n=14)	CHO+W3 (n=15)	W3 (n=14)	*P*-value
Age (years)	60.86±10.55 (63)	63.43±8.56 (63.50)	59.93±11.77 (63)	62.71±10.90 (65)	0.797^A^
Males	12 (86)	10 (71)	7 (47)	9 (64)	0.160^Q^
Body weight (kg)	72.71±12.58 (71.9)	72.82±11.21 (71.2)	65.95±10.69 (66)	73.78±18.66 (69.2)	0.513^K^
Height (m)	1.67±0.10 (1.64)	1.63±0.08 (1.62)	1.64±0.07 (1.64)	1.64±0.10 (1.64)	0.663^A^
BMI (kg/m^2^)	26.26±3.59 (26.6)	26.49±3.38 (26.3)	25.27±3.48 (25.4)	27.26±5.64 (25.7)	0.911^K^
Waist circumference (cm)	92.32±8.30 (90)	97.71±8.55 (97.5)	91.50±12.56 (91)	97.21±13 (97.5)	0.295^A^
Hip circumference (cm)	95.00±5.72 (96)	95.50±6.65 (93)	95.03±11.04 (96.5)	99.50±11.91 (101)	0.625^K^
Waist-hip ratio	0.95±0.09 (0.9)	0.99±0.09 (1)	0.97±0.08 (1)	1.00±0.09 (1)	0.361^A^
SGA A. number (%)	11 (79)	13 (93)	13 (87)	13 (93)	0.617^Q^

A ANOVA;

K Kruskal-Wallis;

Q Chi-square

BMI=body mass index; CHO group = fasting abbreviation with carbohydrate; CHO+W3 group = fasting abbreviation with carbohydrate and perioperative infusion of ω-3 PUFA; Control group = fasting abbreviation with water; n=number; *P*=probability; SGA=subjective global assessment; W3 group = fasting abbreviation with water and perioperative infusion of ω-3 PUFA Data expressed as mean ± standard deviation; median in parentheses for continuous variables or number (%) for categorical variables.

**Table 2:** Clinical data and risk factors for coronary disease in 57 patients undergoing on-pump CABG.

	CHO (n=14)	Control (n=14)	CHO+W3 (n=15)	W3 (n=14)	*P*-value
LVEF	0.55±0.12 (0.60)	0.57±0.11 (0.60)	0.54±0.13 (0.60)	0.62±0.11 (0.65)	0.168^K^
Smoking	6 (43)	9 (64)	7 (47)	8 (57)	0.653^Q^
Diabetes	4 (29)	3 (21)	5 (33)	6 (43)	0.666^Q^
Dyslipidemia	11 (79)	12 (86)	8 (53)	11 (79)	0.204^Q^
Previous AMI	7 (50)	9 (64)	6 (40)	7 (50)	0.631^Q^
Use of beta blockers	9 (64)	10 (71)	6 (40)	11 (79)	0.151^Q^
Use of statins	11 (79)	11 (79)	9 (60)	11 (79)	0.580^Q^
Use of fibrates	2 (14)	2 (14)	2 (13)	3 (21)	0.930^Q^
Previous angioplasty	1 (7)	4 (29)	4 (27)	3 (21)	0.495^Q^

K Kruskal-Wallis;

Q Chi-square

AMI=acute myocardial infarction; CHO group = fasting abbreviation with carbohydrate; CHO+W3 group = fasting abbreviation with carbohydrate and perioperative infusion of ω-3 PUFA; Control group = fasting abbreviation with water; LVEF=left ventricular ejection fraction; n=number; P=probability; W3 group = fasting abbreviation with water and perioperative infusion of ω-3 PUFA Data expressed as mean ± standard deviation; median in parentheses for continuous variables or number (%) for categorical variables.

### Intraoperative Period

There was no incidence of bronchial aspiration during orotracheal intubation nor after anesthesia had worn off. No deaths occurred in the operating room. Intraoperative data are shown in Table 3. There was no statistically significant difference among groups (*P*>0.05).

**Table 3:** Intraoperative clinical data in 57 patients undergoing CABG with CPB.

	CHO (n=14)	Control (n=14)	CHO+W3 (n=15)	W3 (n=14)	*P*-value
Total duration of surgery (min)	244.64±40.31 (240)	232.31±49.27 (225)	249.29±24.01 (240)	253.93±40.86 (270)	0.532^A^
CPB time (min)	77.14±20.37 (82.5)	66.64±24.50 (60)	63.53±15.63 (66)	78.79±22.93 (85)	0.147^A^
CC time (min)	63.71±17.29 (66.5)	56.86±28.21 (50.5)	54.20±15.52 (56)	61.57±18.58 (68)	0.586^A^
Need for transfusion	7 (50)	7 (50)	7 (47)	8 (57)	0.953^Q^
Number of grafts	3.00±0.96 (3)	2.36±0.93 (2)	2.67±0.82 (3)	2.86±0.86 (3)	0.266^A^
Complications	1 (7)	2 (14)	0 (0)	1 (7)	0.519^Q^

A ANOVA;

Q Chi-Square

CC=cross-clamp; CPB=cardiopulmonary bypass; CHO group=fasting abbreviation with carbohydrate; CHO+W3 group=fasting abbreviation with carbohydrate and perioperative infusion of ω-3 PUFA; Control group=fasting abbreviation with water; n=number; P=probability; W3 group=fasting abbreviation with water and perioperative infusion of ω-3 PUFA Data expressed as mean ± standard deviation; median in parentheses for continuous variables or number (%) for categorical variables.

**Fig. 3:**
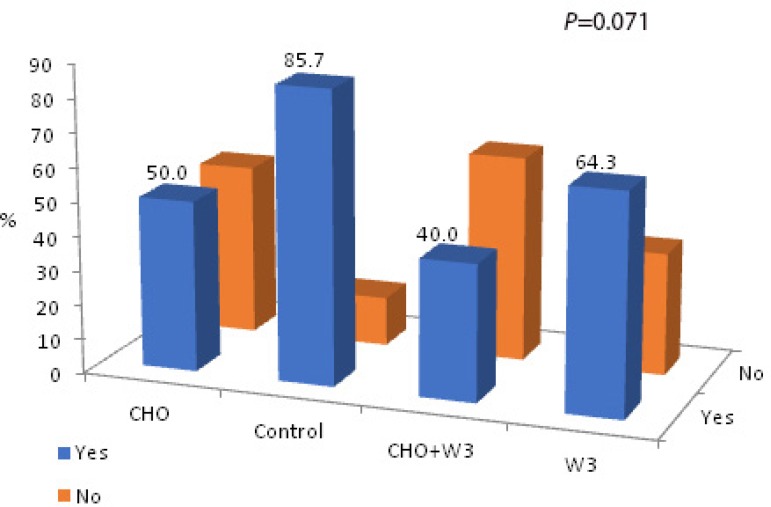
Group distributions of vasoactive drugs required for weaning from CPB.

Although the number of patients who needed vasoactive drugs (dobutamine and/or norepinephrine) for weaning from CPB was higher in the Control group, no significant difference per group was found (CHO: 7/14, 50.0%; Control: 12/14, 85.7%; CHO+W3: 6/15, 40.0%; W3: 9/14, 64.3%; *P*=0.071) (Figure 3). Patients in groups with fasting abbreviation and CHO intake (CHO and CHO+W3 groups) needed fewer vasoactive drugs compared to the other groups (RR=0.60; 95% CI: 0.38-0.94; *P*=0.020) (*P*=0.035).

### Postoperative Period

Overall mean ICU length of stay was 3.11±1.88 days (2-14), with no significant difference shown in means ± standard deviations per group (*P*=0.713), as follows: CHO group, 2.86±1.23 days (median: 2); Control group, 3.43±3.16 days (median: 2); CHO+W3 group, 2.80±0.94 days (median: 3); and W3 group, 3.36±1.55 days (median: 3). Likewise, there was no significant difference in total postoperative length of hospital stay among groups, with an overall mean of 7.75±3.62 days observed (*P*=0.980).

**Fig. 4:**
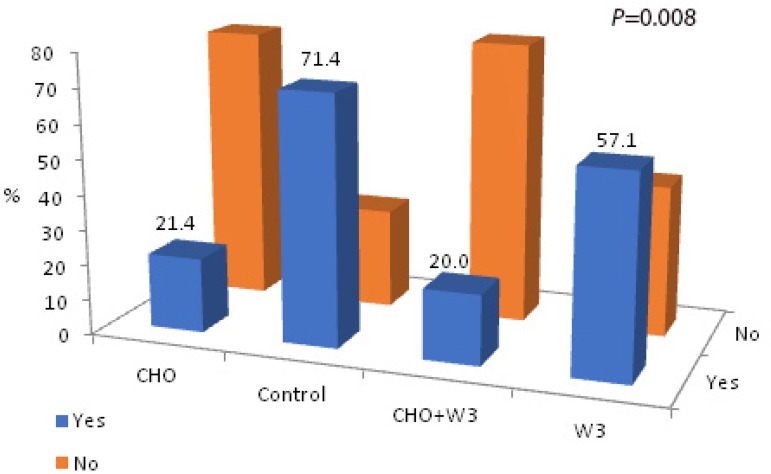
Group distributions of vasoactive drugs required during ICU recovery.

The number of patients requiring vasoactive drugs (dobutamine and/or norepinephrine) during recovery in the ICU differed significantly among the groups (CHO, 3/14; 21.4%; Control, 10/14; 71.4%; CHO+W3, 3/15; 20.0%; W3, 8/14; 57.1%; *P*=0.008). Patients receiving CHO (CHO and CHO+W3 groups) had substantially lower needs for these drugs compared to the other groups (Figure 4).

Incidence of POAF per group differed statistically (*P*=0.009), with overall incidence of 29.8% (17 patients). Six patients (42.9%) presented POAF in the CHO group, eight (57.1%) in the Control group, one (6.7%) in the CHO+W3 group, and two (14.3%) in the W3 group. A significantly lower incidence of POAF was observed in patients who received ω-3 PUFA (CHO+W3 and W3 groups) (*P*=0.001): only three patients (10.3%) receiving ω-3 PUFA developed POAF, the other 14 (50.0%) belonging to the other two groups. Thus, 50.0% of the patients who did not receive ω-3 PUFA developed POAF, a percentage 4.83 times higher than the groups that received it (RR=4.83; 95% CI: 1.56-15.02).

The amount of bleeding in the first 12 hours of recovery in the ICU was 266.92±214.51 mL, with a median of 200±86 mL. There was no statistically significant difference among groups (*P*=0.351). The use of packed red blood cells or blood components did not differ among groups in the operating room and in the ICU (*P*>0.05).

### Infectious Complications

The overall incidence of infectious complications in the ICU was 7.0% (4 patients). All four patients were diagnosed with postoperative pneumonia, two patients from the W3 group and one patient each from the CHO and Control groups, without statistical difference among groups (*P*=0.519). There were no cases of either surgical wound infections or mediastinitis in the ICU. No statistical difference was observed in the number of patients diagnosed with any type of infection during recovery in the hospital ward (*P*=0.179). There was a superficial infection of the saphenectomy wound in two patients from the W3 group and in another two from the Control group, and one of the last patients also had an infection in the lower third of the thoracic surgical wound (median incision). One patient from the W3 group, two from the CHO group, and another two from the Control group had pneumonia during the hospital ward recovery.

None of the patients developed sepsis or mediastinitis in the ward. Considering the incidence of infectious complications per group during the entire postoperative period, no statistically significant difference between groups was found (*P*=0.069), even though the *P* value was close to statistical significance due to the lower cases of complications found in patients of the groups receiving CHO (CHO and CHO+W3 groups) (RR=0.29; 95% CI: 0.09-0.94; *P*=0.023).

### Incidence of Combined Major Cardiovascular Events

Postoperative CVA occurred in 5.3% of the patients (3 patients). Two of the patients were in the ICU: one, from the Control group, had early CVA and died; the other, from the CHO+W3 group, had late CVA and developed left-sided hemiplegia, with partial recovery at hospital discharge. The third patient, also from the Control group, was diagnosed with transient ischemic attack in the hospital ward and was discharged without sequelae. Two patients (3.5%) presented postoperative AMI during recovery in the ICU, one from the CHO and the other from the W3 group. The patient from the W3 group died and there were no repercussions from this event in the patient from the Control group.

Therefore, as mentioned above, two deaths (3.5%) of patients from the Control and W3 groups were recorded, both in ICU recovery and with no statistical difference between the groups (*P*=0.543). In addition, two patients developed postoperative AMI and three patients suffered a CVA. The incidence of combined events (CVA, AMI, and Mortality) was not statistically significant (*P*>0.05).

### Blood Glucose Levels and Insulin Resistance

There were no statistically significant differences between groups for the three capillary blood glucose serial tests performed intraoperatively (*P*=0.425). However, when analyzing the isolated and progressive behavior of these tests in groups of patients who were did not receive CHO (*i.e*., Control and W3 groups), a significant increase was observed in the means of the second and third tests compared to values at the beginning of surgery (*P*=0.022). Of the total number of patients assessed, 77.2% (44) required intraoperative exogenous insulin, in equal amounts and equally distributed across the groups *(P*>0.05).

**Fig. 5:**
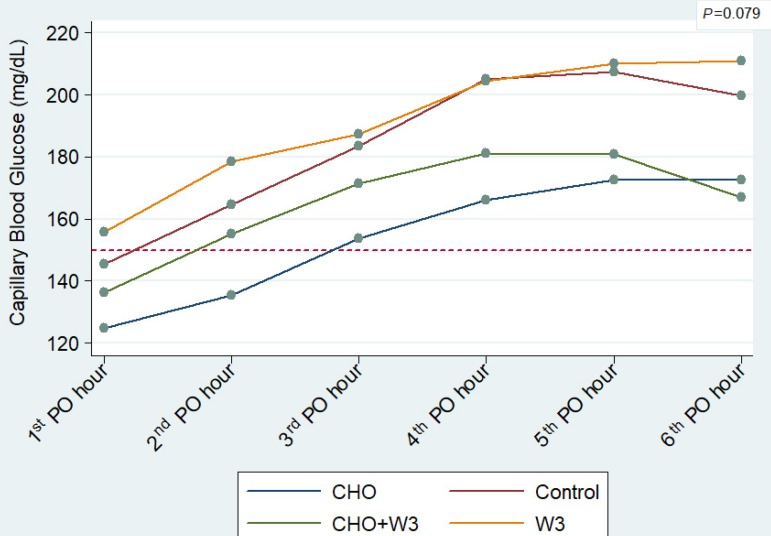
Average capillary blood glucose levels during the first 6 postoperative hours in the ICU, according to groups. Note: the dotted line indicates the 150 mg/dL limit and the regular use of exogenous insulin.

**Fig. 6:**
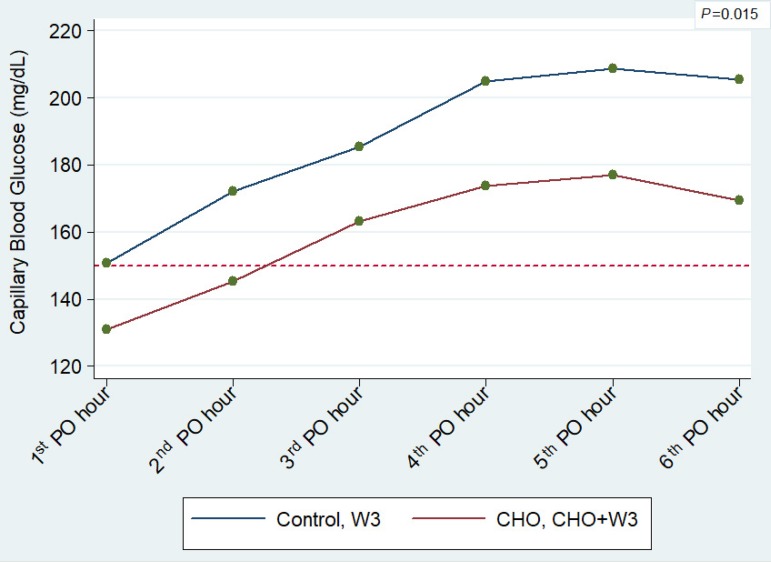
Average capillary blood glucose levels during the first 6 postoperative hours in the ICU, according to a new grouping: fasting abbreviation with CHO (CHO and CHO+W3) vs. groups receiving abbreviation with water (Control and W3). Note: the dotted line indicates the 150 mg/dL limit and the regular use of exogenous insulin.

Likewise, there was no significant difference between the groups for the capillary blood glucose serial tests performed in the first six hours of recovery in the ICU (*P*=0.079) (Figure 5). Nevertheless, it was observed that both groups with patients receiving CHO (CHO and CHO+W3) tended to have better glycemic control. Based on this pattern, results of these two groups (CHO and CHO+W3) were combined and compared with each other (Control and W3). With this new clustering of data, a statistically significant difference was found between the two new clusters (*P*=0.015), showing better glycemic control in groups with brief fasting and CHO intake (Figure 6).

Only four patients (7.0%) did not need exogenous insulin in the ICU to control glycemia. Overall mean for insulin use in the ICU was 22.67±13.36 IU in six hours. On average, the CHO group needed 16±14.38 IU; the Control group, 25.43±11.62 IU; the CHO+W3 group, 19.0±9.59 IU; and the W3 group, 30.29±14.01 IU. The difference between groups was statistically significant (*P*=0.018) due to the low use of insulin for glycemic control in patients who received CHO.

### Inflammatory Response - Inflammatory Markers

**Fig. 7:**
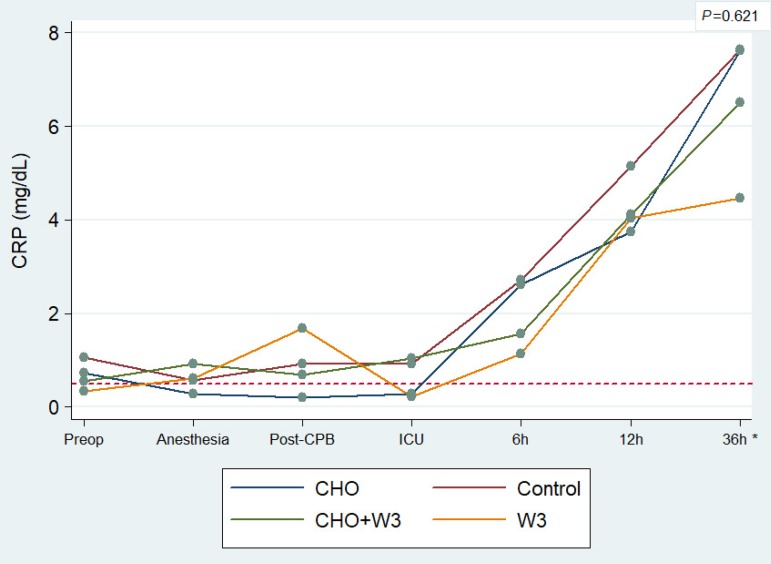
Average level of the CRP inflammatory marker per group, taken from seven serial blood tests. *Refers to case-by-case analysis between groups at 36 hours postoperatively (P=0.008). Note: the dotted line indicates the reference value for acute inflammation used by the laboratory.

Regardless of which group they belonged to, all patients presented a significant increase in ultrasensitive CRP in the serial tests conducted over time (*P*<0.001). This increase occurred mainly after the 4^th^ test, which corresponds to the postoperative period or arrival at the ICU. No statistical difference between groups was found (*P*=0.621); however, in the 7^th^ test (36h PO), mean CRP levels were significantly lower in the W3 group (mean: 4.46±3.37 mg/dL) than in the other groups (mean: 7.24±7.40 mg/dL) (*P*=0.008) (Figure 7).

**Fig. 8:**
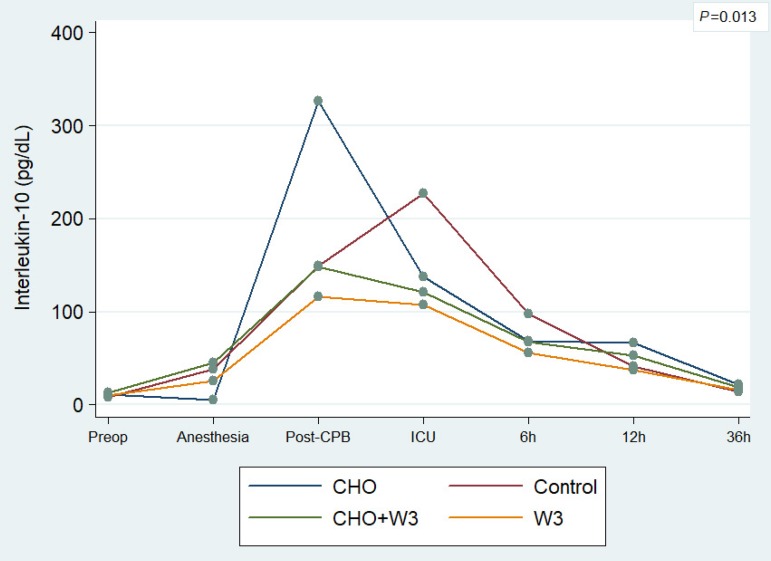
Average level of the IL-10 inflammatory marker per group, taken from seven serial blood tests.

Regarding interleukins, there was no statistical difference between the groups for IL-6 (*P*=0.105), due to the large range of variation and standard deviation found. On the other hand, the results for IL-10 showed a significant association between groups and serial tests (*P*=0.013) (Figure 8). In addition, it was observed that groups of patients who received ω-3 PUFA (CHO+W3 and W3 groups) showed a significantly lower drop (*P*=0.049) in mean values of postoperative recovery period (ICU, 6h, 12h, and 36h), when compared to the groups that did not receive it.

## DISCUSSION

Overall, results show that abbreviation of preoperative fasting with CHO intake associated with intravenous ω-3 PUFA is safe and adequate for patients undergoing on-pump CABG. Not only because patients needed less vasoactive drugs during weaning from CPB or ICU recovery, but also due to lower incidence of POAF, improved metabolic control, and reduced postoperative inflammatory response.

Multimodal protocols are used globally in cardiovascular surgery, and indeed are a recommended practice in CABG guidelines^[5,18,19]^. De Vries et al.^[1]^ compared 3,760 patients who underwent CABG before the implementation of a checklist protocol for 3,820 patients undergoing the procedure after implementation. The number of complications per 100 patients was reduced from 27.3 (95% CI: 25.9-28.7) to 16.7 (95% CI: 15.6-17.9) and the percentage of patients with one or more complications dropped from 15.4% to 10.6% (*P*<0.001). Moreover, there was a reduction in hospital mortality. In Brazil, several centers are concerned with creating, implementing and adapting multimodal protocols - *i.e*., establishing a Heart Team - in addition to maintaining databases in order to evaluate results and adopt measures for general improvement in the services provided^[20,21]^.

Similarly to studies related to the ACERTO Project, the results of this study did not show cases of bronchial aspiration in groups that received CHO and in those who received only water^[2,3,6,14]^. Furthermore, published studies have stated the safety of using ω-3 PUFA in a range of surgical fields in spite of possible effects of oil on platelet function^[22]^.

The outcomes of this protocol are comparable with two previous studies that showed faster postoperative recovery and less need for vasoactive drugs in patients who underwent fasting abbreviation with CHO intake before cardiovascular surgery^[15,16]^. In fact, abbreviation of fasting and CHO intake is nothing new; several centers have stopped practicing prolonged fasting, including for patients undergoing cardiovascular surgery^[17,23]^. Brief fasting reduces insulin resistance, improves metabolic control, and relieves hunger and thirst in the postoperative period^[2,3]^. In cardiovascular surgery, there are still few studies that corroborate these findings; therefore, the results of this study are particularly relevant.

Glycemic and metabolic control is a unique aspect in cardiovascular surgery as it is closely linked to possible clinical outcomes and morbidity and mortality during hospitalization^[24]^. Furnary et al.^[25]^ analyzed 4,864 patients undergoing cardiovascular surgery, in which perioperative hyperglycemia was associated with higher rates of mediastinitis, prolonged hospital stay, and increased costs. In a recent study, Jarvela et al.^[26]^ assessed 1,356 patients undergoing cardiovascular surgery with tight control of repetitive glucose spikes (which can occur in the perioperative period). The authors stated that it occurred in 39.7% of the patients and was associated with higher rates of infection, 12.1% *vs*. 8.2% (*P*=0.019), CVA, 4.9% *vs*. 1.5% (*P*<0.001), and mortality, 6.1% *vs*. 2.1% (*P*<0.001), when compared with patients with blood glucose levels within the normal range or slightly higher.

Much has been discussed about glycemic control and insulin therapy in cardiovascular surgery. Researchers have sought target values - *e.g*., 180 mg/dL, less than 150 mg/dL, or even tighter at 110 mg/dL - to better control metabolism and insulin resistance^[27,28]^. In a recent meta-analysis of 36 randomized studies involving 17,996 patients, Yamada et al.^[29]^assessed four different types of insulin therapy regimen (very mild, mild, moderate, and tight) to treat hyperglycemia in critically ill patients. Their findings indicate that tighter control does not lower the risk of short-term mortality when compared to the very mild regimen [tight (RR=0.94; 95% CI 0.83-1.07; *P*=0.36); moderate (RR=1.1; 95% CI: 0.66-1.84; *P*=0.72); and mild (RR=0.88; 95% CI: 0.73-1.06; *P*=0.18)]. In addition, tight control led to a higher occurrence of hypoglycemia. In a multicenter study, Székely et al.^[30]^ studied the relationship between perioperative hyperglycemia and hospital mortality in 5,050 patients undergoing CABG in 70 cardiovascular surgery centers. Even though mortality was higher among diabetics (4.2% *vs*. 2.95%; *P*=0.02), hyperglycemia was not associated with a higher risk of hospital mortality in these patients. However, in non-diabetic patients, repetitive postoperative glucose spikes between 250-300 mg/dL (RR=2.56; 95% CI: 1.18-5.57; *P*=0.02) and aggressive use of exogenous insulin postoperatively (RR=2.04; 95% CI: 1.12-3.70; *P*=0.01) were considered independent risk factors for mortality.

The results of this study show that abbreviation of fasting with CHO intake interfered in the glycemic control, mainly in the ICU. In the Control and W3 groups, mean capillary blood glucose levels were higher than the pre-established limit of 150 mg/dL starting from the first measurements in the ICU (*P*<0.05), which indicates that closer attention and insulin therapy intervention are needed in these groups to achieve better metabolic control. To test this association, CHO and CHO+W3 groups were clustered and compared to the other two groups (Control and W3). This new grouping showed a statistically significant difference between the groups (*P*=0.015) (Figure 6). Furthermore, the mean overall use of insulin in the ICU was significantly lower in groups that received CHO in comparison with the other*s (P*=0.018).

In this study, insulin resistance was assessed through the need for exogenous insulin as well as oscillations in blood glucose levels postoperatively. This condition may be aggravated by CPB^[31]^. Blood glucose levels tend to increase during hypothermic CPB, whereas insulin levels tend to decline. On the other hand, insulin levels fluctuate and may increase substantially during rewarming, in preparation for weaning from CPB; catecholamines, cytokines, cortisol, and GH levels also increase^[7,16]^.

Inflammation is another relevant issue in cardiovascular surgery. On-pump CABG may trigger the development of SIRS and activation of the complement system, which worsens insulin resistance^[31]^. The patient's blood in contact with non-biocompatible surfaces of the CPB circuit, surgical trauma, and reperfusion injury caused by the method have been considered precise mechanisms for this event. Restoring blood flow at the end of CPB (reperfusion) may worsen lesions caused by ischemia, leading to irreversible injury, release of inflammatory mediators, and apoptosis. Reintroduction of molecular oxygen in ischemic tissue produces oxygen-free radicals, which are harmful to cells and may induce acute systemic inflammatory response^[8]^.

Regular perioperative use of agents such as corticosteroids and mannitol, among others, has been evaluated and some studies have shown its benefits towards minimizing SIRS after CPB^[7,32]^. Although this remains a controversial topic and not yet completely defined in the literature, patients in this study received corticosteroids during induction of anesthesia and mannitol during CPB. Indeed, in a recent publication (European guideline), its routine use in patients undergoing cardiovascular surgery is no longer recommended^[33]^.

A reasonable alternative, currently under discussion for cardiovascular surgery, is the use of pre- or perioperative ω-3 PUFA. Berger et al.^[11]^carried out a randomized placebo-controlled study to assess some inflammatory markers in two groups: the first group received ω-3 PUFA (0.2 g/kg) infusions 12 and 2 hours preoperatively and immediately after surgery; the other received saline solution as placebo (control). There was a significant decrease in postoperative IL-6 (*P*=0.018) and IL-8 (*P*=0.005) in the ω-3 PUFA group, in addition to a reduction in the incidence of arrhythmia, though the latter was not statistically significant. Additionally, plasma concentrations of glucose, lactate, and carboxyhemoglobin were also lower in the intervention group compared to the control group (*P*<0.05).

The benefits of ω-3 PUFA have been reported for decades in several medical specialties, particularly in cardiology and intensive care. Intravenous ω-3 PUFA is rapidly incorporated by cell membranes^[10]^ and may minimize the production of pro-inflammatory mediators^[11]^. These anti-inflammatory properties may lead to shorter hospitalizations and fewer severe infections in critically ill patients. They have also been associated with a lower incidence of postoperative complications and possibly lower mortality from acute lung injury^[34]^. In cardiology, benefits include less morbidity and mortality from congestive heart failure and lower mortality rate from sudden death after AMI^[35]^. Use of ω-3 PUFA has also been linked to better control of dyslipidemia, heart rate, and chronic atrial fibrillation^[36]^. Nonetheless, the literature is conflicting since some studies have not confirmed those benefits^[37]^.

The protocol in this study has shown lower incidence of POAF, in accordance with recent findings from a meta-analysis by Langlois et al.^[12]^. The authors described a systematic review of 19 randomized clinical trials with 4,335 patients who underwent cardiovascular surgery. Similarly to what was observed in our study, the meta-analysis could not identify the effect of ω-3 PUFA on ICU length of stay and postoperative mortality. However, infusion of ω-3 PUFA was associated with shorter hospital stay and lower POAF rate, especially with CPB. Reduced POAF observed in our patients and in the meta-analysis described above may likely be related to the lower postoperative inflammatory response as a result of the infusion of ω-3 PUFA, although we know that POAF mechanisms have multiple factors and are different from those found in paroxysmal atrial fibrillation^[12]^.

This study has also shown interesting findings related to postoperative inflammatory response and ω-3 PUFA. IL-10, the only cytokine with measured anti-inflammatory properties, was statistically different per group (*P*=0.013) and remained higher postoperatively in patients who received ω-3 PUFA (*P*=0.049). Likewise, patients from the W3 group, who received only ω-3 PUFA, had lower levels of ultrasensitive C-reactive protein (PCR) within 36 hours postoperatively (*P*=0.008).

The findings presented in this study must be interpreted with caution since the sample size was a limiting factor. Additional studies about the issues presented here are warranted given the promising effects of both interventions on metabolism and postoperative inflammation.

## CONCLUSION

From the results observed, it is possible to conclude that the abbreviation of fasting with CHO associated with perioperative infusion of ω-3 PUFA is safe and supports faster postoperative recovery in patients undergoing on-pump CABG.

**Table:** 

Authors' roles & responsibilities	
GRF	Conception and design of the work; analysis and interpretation of data for the work. Drafting the manuscript and revising it critically for important intellectual content; final approval of the version to be published
PRLL	Acquisition of data; final approval of the version to be published
ACF	Acquisition of data; final approval of the version to be published
FRHLC	Acquisition of data; final approval of the version to be published
DCB	Acquisition of data; final approval of the version to be published
LRT	Acquisition of data; randomization of the study; final approval of the version to be published
NJS	Drafting the manuscript and revising it critically for important intellectual content; final approval of the version to be published
JEAN	Drafting the manuscript and revising it critically for important intellectual content; final approval of the version to be published
